# An interview with Marissa C. Keesler

**DOI:** 10.1590/2176-9451.19.2.027-038.int

**Published:** 2014

**Authors:** Fernanda Catharino, Luciana Q. Closs, Nelson Mucha, Roberto Lima

**Affiliations:** Professor, Department of Orthodontics, Bahiana School of Dentistry. » Professor at the Specialization course in Orthodontics, Federal University of Bahia (UFBA). » Certified by the Brazilian Board of Orthodontics and Facial Orthopedics (BBO). » MSc in Orthodontics, Federal University of Rio de Janeiro (UFRJ). » PhD resident in Orthodontics, UFRJ.; Coordinator of the Orthodontic post-graduate program and professor at the specialization and undergraduate programs, Lutheran University of Brazil (ULBRA). » Certified by the American Board of Orthodontics (ABO) and by the Brazilian Board of Orthodontics and Facial Orthopedics (BBO). » MSc in Orthodontics, University of Detroit Mercy. » PhD in Orthodontics, State University of São Paulo (UNESP)/Araraquara.; Full professor, Department of Orthodontics, School of Dentistry, Fluminense Federal University (UFF). » Visiting professor, University of Passo Fundo and USP/Ribeirão Preto. » MSc and PhD in Orthodontics, UFRJ.; Former director of the Brazilian Board of Orthodontics and Facial Orthopedics (BBO). » Certified by the American Board of Orthodontics (ABO). » Specialist, University of Illinois, USA. » MSc and PhD in Dentistry, UFRJ.

## Abstract

Dr. Marissa Keesler attended dental school at Creighton University in Omaha, Nebraska and in 1987 received her Doctor of Dental Surgery degree with high honors. In 1989, she graduated from Marquette University with a Certificate and Master of Science degree in Orthodontics. Dr. Keesler has been an Adjunct Professor in the Marquette Graduate Orthodontic Department since 1990 and has been a guest speaker at several universities and orthodontic groups nationally and internationally. She has contributed to the development of several orthodontic textbooks in topics related to multidisciplinary treatment and the indirect bonding technique. Dr. Keesler is a member of the American Association of Orthodontists and many other local and national dental and orthodontic societies such as the Edward H. Angle Society of Orthodontists and the Pierre Fauchard Academy. She also has Diplomate status with the American Board of Orthodontics and is a graduate of the AEO Roth/Williams Center. Dr. Keesler has been in specialty practice since 1989 and has had a full-time private practice in Neenah since 1992. In 2000, she was joined by her husband, Dr. Jeffrey T. Keesler, who has a dual specialty in Prosthodontics and Orthodontics. Dr. Roberto Lima Filho

## Abstract (pt)

A Dra. Marissa Keesler graduou-se em Odontologia pela Creighton University (EUA) e em 1987 recebeu, com distinção, o título de Doutora em Odontologia, área de concentração em Cirurgia. Em 1989, recebeu o título de Mestre em Ortodontia, pela Marquette University. É professora adjunta no Curso de Graduação do Departamento de Ortodontia da Marquette University desde 1990, além de conferencista convidada em várias universidades e grupos relacionados à Ortodontia nos EUA e ao redor do mundo. Tem dado valiosa contribuição na publicação de diversos livros, relacionados ao tratamento multidisciplinar e à técnica de colagem indireta. É membro de renomadas entidades científicas como a Associação Americana de Ortodontistas, Sociedade Edward H. Angle, Academia Pierre Fauchard, entre outras. Diplomada pelo conceituado American Board of Orthodontics e graduada pelo AEO Roth/Williams Center, a Dra. Keesler está envolvida na prática de especialidades odontológicas desde 1989 e tem trabalhado em sua clínica particular, na cidade de Neenah (Wisconsin, EUA), desde 1992. Dr. Roberto Lima Filho

**Figure:**
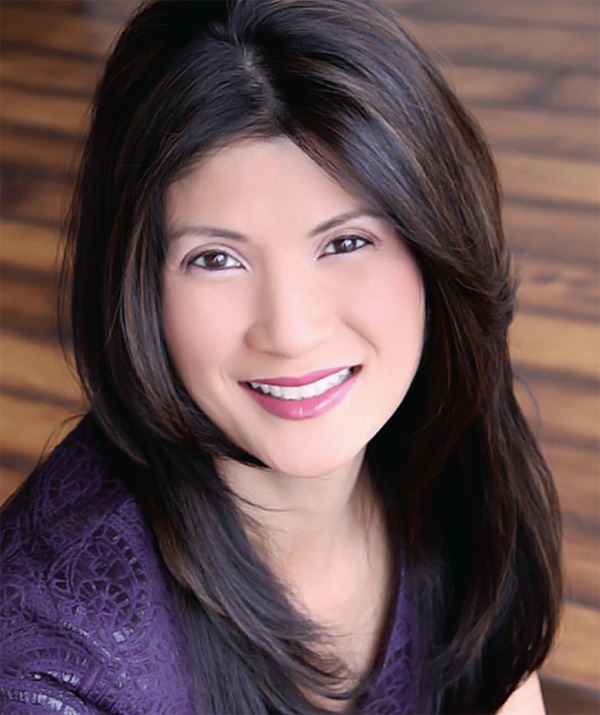


## What differences in treatment efficiency do you notice in your clinical practice between patients who undergo 2 -phase treatment and those who do not? Fernanda Catharino

The question that I routinely ask myself when determining whether early orthodontic treatment should be considered or not is, "Can the problems presented by this young patient worsen as we wait for the rest of the deciduous teeth to exfoliate? Or, can they remain the same or perhaps even improve naturally?" If it is the first of the two, then early treatment is recommended. Some of the problems corrected during this first phase may include moderate to severe crowding, deleterious habits (e.g., thumb or tongue), ectopic eruption of certain permanent teeth (e.g., maxillary first permanent molars), crossbite of permanent teeth, and functional crossbite of deciduous teeth. I try to time the start of all Phase I treatments adequately so that the duration can be kept within one year or less. Parents are made aware that after all the permanent teeth erupt, new diagnostic records will be gathered to determine the need for a second phase of treatment. If indicated, Phase II can oftentimes last anywhere between 12-18 months depending on the malocclusion and skeletal discrepancies remaining.

If a single phase of comprehensive treatment is more advisable for the young patient, growth and development is monitored every 6-12 months until all the permanent teeth have erupted, including the second molars. Waiting until the second molars erupt to start comprehensive treatment will ensure that any eruption and torque problems presented by these teeth can be addressed immediately and that treatment will progress quickly without having to scale back down in archwire size later, or have to do additional treatment in the future. Comprehensive therapy can last anywhere between 12-24 months depending on the severity of the problems.

While it is true that a single phase of treatment may seem more efficient in terms of cost and the final sum of treatment time, I believe that there are many cases where the final outcome, including facial and dental aesthetics and periodontal health, can be more ideal with two phases of treatment.

## Regarding the effects of early intervention on jaw growth, what is the optimal time to start treatment in Class II patients? Fernanda Catharino

I mostly tend to use the dentitional stage (late mixed or early permanent dentition) to determine the optimal time to start Class II therapy. I also follow the cervical vertebra maturation (CVM) method developed by Don Lamparski^1,2^ and advocated by Jim McNamara. The maturational stage is determined by the level of cervical vertebra maturation observed in the lateral headfilm and the most effective time seems to be during the circumpubertal growth period.

Most of the time, if the discrepancy in centric relation is less than half a tooth, a headgear will be considered. Those that are end-to-end or larger are usually treated with a fixed Herbst appliance, keeping in mind that most studies have shown that the treatment effect produced by this tooth-borne type of functional appliance is 50% dental and 50% skeletal.^3,4^ Careful attention is also paid to cephalometric evaluations that indicate whether mandibular growth will be favorable or not (e.g., Jarabak's upper and lower gonial angles, and mandibular corpus length).^5^ When Class II skeletal discrepancy is severe and mandibular growth is not favorable, growth and development is monitored and surgical-orthodontic treatment is suggested after the patient has matured skeletally.

If the patient presents a severe Class II that may be affecting his or her self-image or self-esteem, intervention may be done earlier with everyone's understanding (including patient, parents, and referring dentist) that comprehensive treatment will still be necessary later and that this may include orthognathic surgery.

## What are the main aspects that should be observed in the finalization of orthodontic treatment that, in your opinion, contribute to long-term stability? Fernanda Catharino

While it may be argued whether non-ideal occlusal relationships have an impact on the dentition,^6^ it is my personal opinion, after evaluating our finished cases, that long-term stability can be influenced by the attainment of an ideal functional occlusion, the amount of CR/CO discrepancy remaining, and the amount of occlusal detailing that is accomplished by the end of treatment. Excellent bracket placement since the start of treatment will minimize archwire bends or re-bonds, thus reducing the amount of roundtripping of the teeth as well as the treatment time. A good retention protocol is also an important factor contributing to the long-term stability of results.

## A problem faced by many clinicians during the indirect bonding technique is the excess resin around the brackets. How can this problem be avoided in the bonding technique you use? Fernanda Catharino

The Indirect Bonding technique is a very sensitive procedure. For this reason, special attention should be paid to every step so that ideal results can be routinely achieved. The following is the protocol used in our practice for bracket placement on working casts.

Using a thin black lead pencil, such as a 0.03 mm Pentel^TM^ pencil, the long axis and marginal ridges of the teeth are drawn on dry working casts. A marking is done 2 mm below the marginal ridge line on all posterior teeth indicating the position of each bracket slot. A bow divider (Dentraurium^TM^) and a millimetric ruler are used to determine the 2 mm marking. This will ensure that all marginal ridges will be leveled at the end of treatment. In the mandibular arch, the distance from the bracket slots to the cusp tips of the first bicuspids is transferred to all the incisors and 0.5 mm is added for the canines. In the maxillary arch, the distance from the bracket slots to the cusp tips of the first bicuspids is transferred to the lateral incisors and 0.5 mm is added for the centrals and the canines.

Two thin coats of a mix of separating medium and water in a 1:6 ratio are applied to the casts and let dry for 5 minutes. A light-cured composite, such as Transbond XT^TM^ (3M Unitek), is placed on the mesh pad of each individual bracket to create the custom base. The brackets are then placed on the working casts. It is important that each bracket is placed as accurately as possible right away to minimize the amount of repositioning done before the composite is cured, thus, maintaining the integrity of the composite custom bases. The brackets are positioned and the excess resin is removed around the periphery of each bracket with the aid of a Hollenbach instrument. The casts are then placed in a Triad^TM^ 2000 light curing unit for 5 minutes.

**Figure 1:**
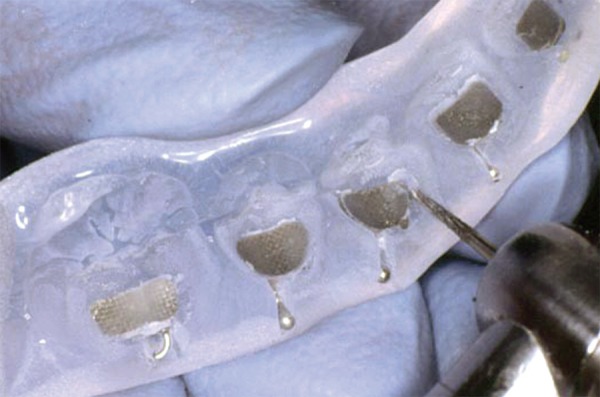
A straight bur on a high speed handpiece can be used to remove any excess composite around the periphery of the custom resin.

There are several different ways in which the custom transfer trays can be fabricated. After trying many different tray systems over the past 26 years (including the two years in my residency program at Marquette University), I have concluded that the dual tray system provides the most accurate results in a consistent manner. A coating of an effective separating medium, such as General Purpose Silicone Mold Release or PAM cooking spray, is sprayed over the casts and brackets and a soft, 1.5 mm sheet of clear Bioplast^TM^ material is then vacu-formed over the bracketed casts in a Biostar^TM^ machine (Great Lakes Orthodontics). The excess material is trimmed using a pair of small scissors. Another coating of separating medium is sprayed over the outer surface of the Bioplast tray and a 1-mm thick layer of Biocryl is then vacu-formed over the soft tray in the Biostar. The separating medium prevents adhesion and allows customization of the two trays. The excess Biocryl material is trimmed with small scissors and the outline of the tray is cut using a slow speed handpiece cutting disc. The cast is submerged in water for approximately 10-15 minutes. The trays containing the brackets are removed from the casts with the aid of a plaster knife and are immediately placed in the Triad unit for an additional 1 minute with the brackets facing upward. The custom resin bases are then micro-etched very lightly with 50-micron aluminum oxide to clean them and to permit a good bond. As a general rule, 1-2 seconds should be enough, making sure that some of the resin is not abraded away. The excess aluminum oxide is removed from the bracket bases using compressed air. The bases are then cleaned with a hand-held steam cleaner. After drying thoroughly, a straight bur on a high speed handpiece is used to remove any excess composite around the periphery of each bracket (Fig 1). The trays are separated and the soft tray is trimmed with small scissors and the hard tray with a sharp Bard-Parker knife. The soft Bioplast tray must extend to the gingival margin, whereas the hard Biocryl tray needs to extend only as far as the bracket slots. The areas around the bracket hooks are relieved on the soft tray by making a simple cut next to each hook with small sharp scissors to allow for easy removal of the tray during the clinical procedure. The soft and hard trays are then put back together.

**Figure 2:**
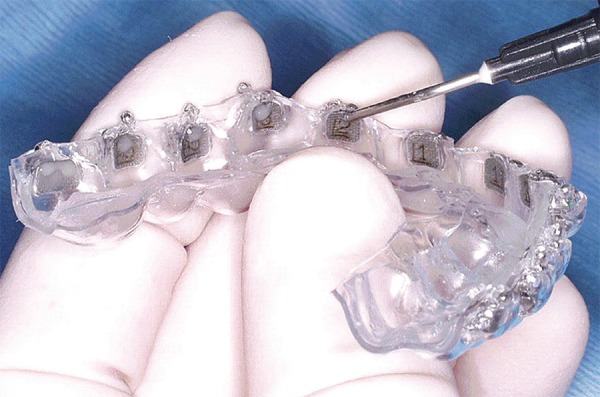
Flowable adhesive is applied along the gingival edges of each custom resin base, a line on the molars and a dot on the rest from second bicuspid to second bicuspid.

The protocol used in our practice for the chair-side bonding procedure is as follows:

After all the tooth surfaces to be bonded are well cleaned, each custom bracket base is prepared with Assure^®^ Universal Bonding Resin (Reliance Orthodontic Products). A thin line of flowable adhesive is applied along the gingival edge of each molar bracket custom resin base, and a small dot is made on the middle area of the gingival edge from second bicuspid to second bicuspid (Fig 2). The prepared indirect bonding trays are placed under a light protective box until they are ready to be placed over the teeth. The oral cavity is well isolated using NeoDrys^TM^, the cheek retractor portion of the Nola Dry Field System (Great Lakes Orthodontics, Ltd.), and the tongue shield portion of the Quest^TM^ Dry Field System (Ortho Quest). The teeth are dried thoroughly. The facial surfaces of all teeth to be bonded are etched on one arch using a viscous liquid or gel 37 or 40% phosphoric acid for 15 to 30 seconds followed by thorough rinsing. Each tooth is well dried using a warm air tooth dryer. Using a brush, a thin layer of Assure Universal Bonding Resin is applied to the etched enamel. The advantage of this moisture insensitive primer is that it can be used on most any surface. The tray is then seated and each tooth is cured for 10 seconds using either a high intensity halogen or LED curing light, or 5 to 10 seconds with an argon laser. The procedure is repeated for the opposite arch. The hard outer tray is removed easily and the soft inner tray is removed with the aid of a scaler to peel away the material from around each bracket wing.

**Figure 3:**
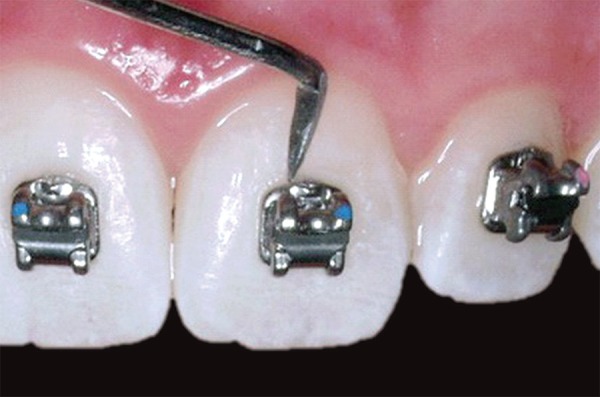
If each step is carried out carefully, minimal to no adhesive flash should remain around each bracket at the end of the indirect bonding procedure.

If all the steps are carried out carefully, the amount of adhesive flash remaining around the brackets at the end of the procedure should be minimal to none and can be easily removed with a scaler (Fig 3).

## In your clinical experience, what are the advantages (besides hygiene) for the use of self-ligating brackets compared to conventional appliances? Luciana Closs

We have used the self-ligating bracket system in our practice for approximately 14 years, and we have been able to experience several advantages that have an impact in the quality of our treatment results compared to the use of conventional brackets. The most important, in my opinion, is the ability to obtain full expression of each bracket prescription with the use of larger stainless steel archwires which can be engaged with ease using "active" self-ligating brackets. A routine sequence of archwires in our practice may consist of: 0.014 NiTi, 0.019 x 0.025-in BioForce^®^ (Dentsply GAC), 0.019 x 0.025-in stainless steel, and 0.022 x 0.028-in stainless steel. It will be difficult to engage and close the doors on every bracket with a larger archwire if the previous one has not been allowed enough time to fully express itself. If this happens, the previous wire will need to be kept in longer or a smaller jump in size of archwire would need to be considered.

In our practice, ideal finished results with proper torque and rotational corrections are consistently obtained using "active" self-ligating brackets without having to rely on the use of steel ties.

These types of brackets and the use of the indirect bonding technique are two important tools that allow us to obtain excellent tooth positions in a reliable and consistent manner.

## What is your protocol for retention? Would you recommend the long-term use of a bonded 3-to-3, based on the risk of ankylosis of the bonded teeth? Luciana Closs

Our protocol for retention consists of the following: a couple of months prior to the end of treatment, CR mounted models are taken to evaluate the occlusion and each individual tooth position in all three dimensions. If minimal additional adjustments are necessary, these are carried out and the same mounted models are sent to a commercial laboratory for the fabrication of a CR tooth positioner. If a mandibular fixed 3-to-3 lingual retainer is part of the retention plan, perhaps due to severe pre-treatment dental rotations or large diastemas, an impression is taken one week prior to removal of the fixed appliances for its fabrication. At the debond appointment, the fixed retainer is indirectly bonded first, followed by bracket and composite removal and aesthetic enhancements. The CR tooth positioner is tried in and instructions are given to the patient (wear full time for the first 4 days followed by 4 hours plus nights for 7 weeks). At the following appointment, post-treatment records are taken as well as an impression for maxillary and mandibular removable retainers. Most of the time, the upper is a clear plastic retainer that will also protect the teeth from any mild clenching or grinding habits. If rapid palatal expansion was part of the treatment, a maxillary wrap around retainer is fabricated instead. A spring aligner is used as the lower removable retainer. The teeth should settle for the most part with the positioner, so the patient is instructed to wear the removable retainers 12 hours every night as soon as they are delivered.

If a mandibular fixed retainer is used, it is left in place for at least 2 years, or until the status of the third molars is determined in adolescents. After this, the patient is given the option of keeping the fixed retainer in place for an indefinite period of time, or of having it replaced with a spring aligner. Almost always, patients will choose to keep the fixed retainer. The option is not offered when calculus tends to build-up easily around the wire. In this case, the fixed retainer is removed automatically and replaced with a removable retainer after 2 years of retention, or prior if the general dentist recommends it.

In my experience, I have never really observed any true ankylosis of bonded teeth being caused by the long-term use of a fixed 3-to-3 retainer. What I have observed, in certain cases, is the relapse of an open bite or of an impacted tooth become more noticeable when the teeth previously affected are splinted together with a fixed retainer.

## What are the indications for Invisalign in your practice and how do you feel about it in finishing your cases? Luciana Closs

Even though Invisalign is available in our practice, it is not routinely my treatment of choice unless it is the only option the patient wants to consider (mostly adults) and I determine that he or she is an appropriate candidate. I will consider Invisalign only in cases with minor crowding or spacing and where occlusal or skeletal discrepancies are not necessary or expected to be corrected.

**Figure 4:**
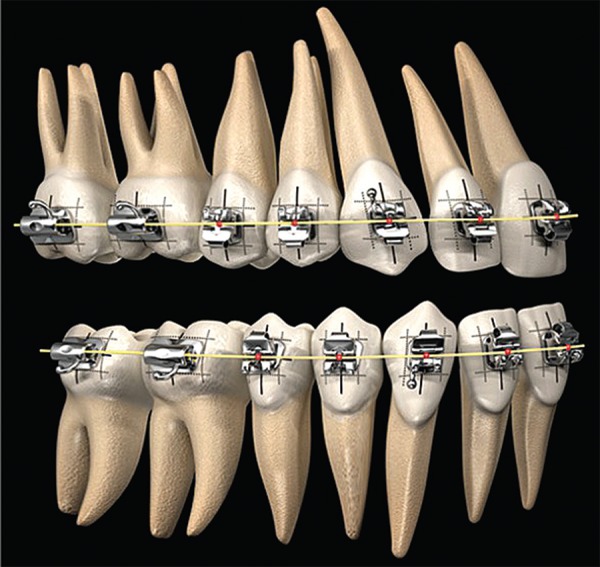
Andrews^®^ Plane: The surface or plane on which the mid transverse plane of every crown in an arch will fall when the teeth are optimally positioned. This plane virtually connects the appliance through the FA point.

With the techniques that we employ in our practice, I have never felt the need to consider Invisalign or other computer-based systems to finish any of my cases. After taking a progress panoramic radiograph to re-evaluate tooth positions mid-treatment, if it is determined that additional detailing is necessary, it will be done with bracket repositions. If the brackets are properly placed on each tooth and an appropriate bracket system is used, every case should be able to be finished with a straight wire using the straight-wire appliance (Fig 4).^7,8^

## What are the tools you use to get compliance during treatment and post-treatment (For instance, the use of elastics and retainers)? Luciana Closs

Good communication and accurate documentation is another aspect of our practice that is critical to achieve our treatment goals and to meet everyone's expectations. Initially, it is imperative to gather accurate information and records, which will yield the best diagnosis and treatment plan for the patient. Stephen Covey, in his highly acclaimed book The Seven Habits of Highly Successful People, suggests that we "Begin with the end in mind". While this is great advice for any endeavor, it is fundamental to orthodontic care. Before treatment is initiated, it is important to have a clear understanding of what the outcome should be to design appropriate treatment goals and be able to share these with the patient, parents, and referring dentist.

A thorough explanation of the importance of the elastics and retainers and possible repercussion with poor cooperation, should be part of the information given to the patient at the time when these appliances are introduced. If poor compliance is detected and addressed and no improvements are noticed up to a couple of appointments after, it is part of our practice policy to immediately take intraoral photographs and share these with the patient and parents during a private consultation at the end of the appointment. Improvements that have been achieved up until then are pointed out and the remaining work necessary to finish treatment is discussed as well as the challenges presented by the lack of cooperation. A time frame to demonstrate improvement is given to the patient. If no changes are observed after the time stipulated, braces are removed and a maxillary splint retainer is given to the patient to be worn every night for an indefinite period of time. The splint will help to protect the teeth and joints and compensate for the remaining malocclusion until the patient decides later in life if he or she wants to have the treatment completed. The cooperation consultation also gives us the opportunity to analyze the family's commitment and desire to continue striving for ideal results. If a negative attitude is observed, removal of the braces is suggested right away as the best alternative to prevent irreversible damages from occurring and additional time from being wasted.

Very few of these consultations are necessary in our practice given that we always try to be proactive instead of reactive from the start of treatment. An excellent diagnosis stemming from the gathering of accurate pre-treatment records can also prevent us from erroneously accusing patients of not cooperating when results are not being observed with certain appliances, such as elastics or headgears, when more likely it could be due to a misdiagnosis and trying to do the impossible.

It is also important to maintain good communication with the referring dentist.

In the 1980's, the sociologist/futurist Avrom King postulated that fine Dentistry was behaviorally self-limited. King meant that regardless of the dentist's technical expertise, unless he or she had the requisite behavioral skills to communicate, including the ability to profoundly listen to his patients and then motivate them, his technical skills would go largely unused.^9^

## Considering the exceptional quality of your finished cases, we would like to know if besides the indirect mounting of brackets, you use some other gimmick at the debonding stage. Nelson Mucha

In my opinion, some of the most important technical aspects that help us to obtain good quality results include: the use of the indirect bonding technique and self-ligating brackets, CR mountings of diagnostic models, evaluating the coordination of upper and lower archwires at each appointment, and the use of centric relation tooth positioners immediately after debond.

If it has not already been done at some point during treatment, the incisal edges of the maxillary and mandibular incisors are reviewed and equilibrated close to debond to confirm that these teeth are at proper vertical heights and that they follow the patient's smile arc. If severe incisal fractures are present, the recommendation for restorations is done immediately following tooth positioner wear. Any type of build-ups of small teeth, such as peg lateral incisors, are done as soon as enough space has been created mesial and distal to these teeth to avoid the need for prolonged use of coil springs.

## Still in relation to finishing, do you use a special procedure sequence or a check list before debonding? If so, what or where would they be? Nelson Mucha

Midway through treatment and prior to the initial placement of a rectangular stainless steel archwire, a progress panoramic radiograph is taken to evaluate root positioning and angulations of the teeth. Keeping in mind the possibility of radiographic distortions, this x-ray is used simply as a guide to decide whether certain brackets need to be repositioned. When it is determined that the patient is ready for debond, a series of appointments are scheduled. The first one consists of an appointment to gather impressions, a hinge axis, and a power centric bite for the fabrication of a tooth positioner. At a second appointment, impressions are taken for any fixed retainers indicated. The last of the series of appointments takes place a week later for the actual debond. The fixed lingual retainers are placed also by means of the indirect bonding technique. Brackets are removed and the CR tooth positioner is tried in and delivered.

The centric relation mounted models gathered for the fabrication of the tooth positioner also allows us the opportunity to do a final evaluation of each tooth prior to debond. Most of the time, no additional adjustments are necessary at this point, otherwise, they are done at the following appointment when the impressions for the fixed lingual retainers are taken. If major discrepancies are noticed, appointments may be reevaluated and discussed with the patient for re-scheduling. At this stage, we will also check that all of our treatment goals, including facial aesthetics, dental aesthetics, functional occlusion, periodontal health, and healthy temporomandibular joints, have been achieved.^10^

## What is the need for anatomical tooth reshaping or occlusal adjustments during the finishing procedure? If there is a need, what procedures are used? Nelson Mucha

Anatomical reshaping or occlusal equilibration, when properly done, can be an important step to further enhance the finished orthodontic results.^11^ It allows the reduction of any remaining CO-CR discrepancy, thus increasing stability of results. Occlusal equilibrations are not always possible to do on every finished comprehensive case, perhaps because the patient may not want to invest any more time and money into their treatment, or because the general dentist may not feel confident enough to perform the procedure. Whenever possible, it is carried out on all non-growing patients post-orthodontics and after the occlusion has settled.

**Figure 5:**
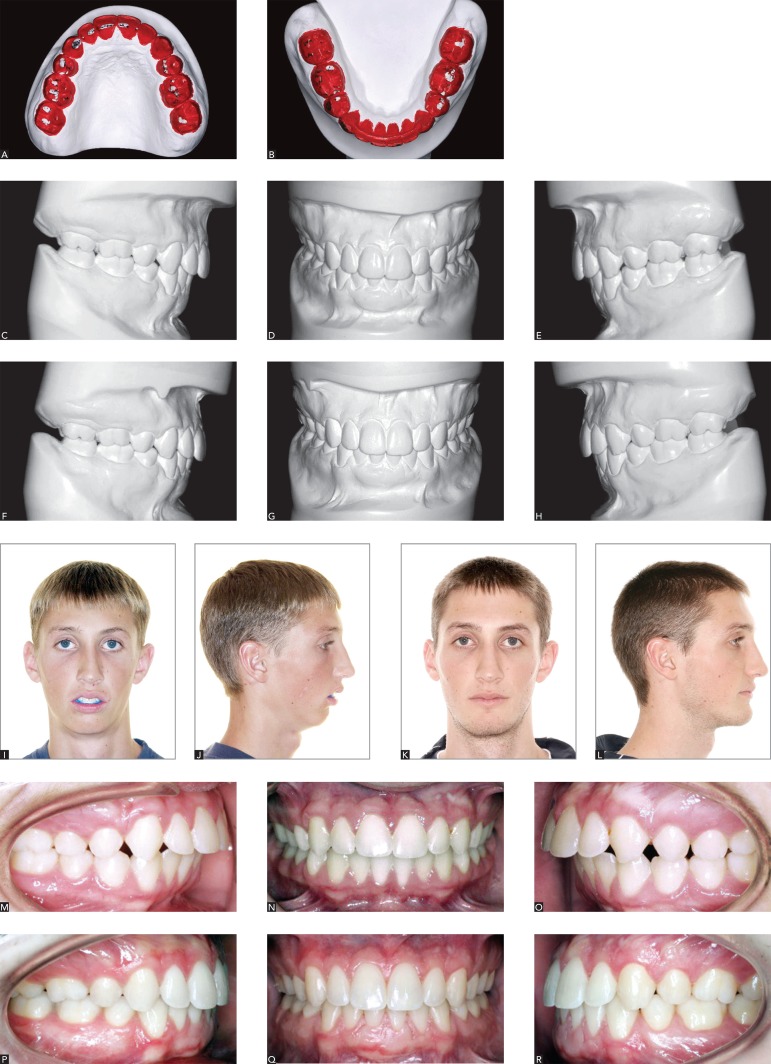
A, B) An occlusal analysis is done initially on CR mounted models before the occlusal equilibration is carried out on the patient. C-H) Comparison of pre (top row) (C, D, E) post (bottom row) occlusal equilibration. F, G, H). Pre-treatment (I, J; M, N, O) and post-treatment facial photographs (K, L; P, Q, R) of the same patient shown in Figures 5A to 5H. The patient had completed comprehensive treatment at a different orthodontic office one month before the pre-treatment photographs were taken. The patient and his family were unhappy with the results and had been referred to us by their general dentist for a second opinion. Four family members were in the dental field and felt that the final occlusion was not ideal. The patient presented with a severe Class II skeletal discrepancy which had been initially misdiagnosed and orthodontically treated only for several years. After gathering the proper diagnostic records, it was decided that a connective tissue grafting procedure to reinforce the attached gingiva on the facial of the mandibular incisors, extractions of the second bicuspids to reduce the proclination of the incisors, and orthognathic surgery would be necessary to attain the most ideal functional occlusion.

When occlusal equilibration is indicated, the patient is placed on a CR splint (preferably maxillary) full-time for a minimum of 3 months to deprogram the muscles. Centric relation records are taken post-splint and the teeth on the models are painted using any red water based paint. A preliminary equilibration is done on these casts (Fig 5A, 5B). The white stone will show up on the areas that have been filed making it easier to recognize them with articulating paper in the patient's mouth so the adjustments can be duplicated. After the equilibration is completed, new CR articulated working casts are gathered and the steps are repeated once or twice more. The patient continues to wear the splint full-time during this time. When all balancing interferences are eliminated, the patient is instructed to wear the splint at night. There are times when negative coronoplasty is not enough to restore the patient back to an ideal functional occlusion and positive coronoplasty, i.e., restoration of teeth may also need to be considered on some of the anterior teeth. "Mastery simply requires we go beyond our limitations to produce results beyond the ordinary." Avrom King.

## As we learn from your lectures, we can see your great knowledge in relation to diagnostic procedures and treatment planning, especially regarding the numerous cephalometric analyses used. Seeking to summarize all this information, which cephalometric data or diagnostic criteria do you consider more important or more relevant for a proper diagnosis and consequently to treatment success? Nelson Mucha

The gathering of quality initial records is important to precisely diagnose, create optimal treatment plans and complete those treatment plans efficiently and effectively. There are times when the patient may not be able or willing to go ahead with the ideal treatment plan recommended (e.g., orthognathic surgery), but it is important to properly inform the patient and be able to offer alternate treatment options with the understanding that the occlusal problems may be improved, but not totally corrected and that a splint may be necessary as an indefinite night retainer to protect the teeth and joints. With all the information being clear to everyone and the expectations being kept consistent, treatment can be carried out efficiently and effectively in the least amount of time possible.

The initial records routinely collected in our practice consist of panoramic and cephalometric radiographs, facial and intraoral digital photographs, CR articulated models, and a CT scan in cases with skeletal asymmetries or where the panoramic radiograph may have indicated anomalies or eruption problems (e.g., impacted or supernumerary teeth). Cephalometric analyses, such as Steinert, Ricketts, and Jarabak, are done, as well as a Visual Treatment Objective (VTO). A VTO will allow us to forecast the normal growth of the patient and the anticipated influences of treatment, to establish the individual objectives we want to achieve for that patient and establish the most ideal treatment mechanics used to reach these goals.^12^

## Do you believe in early treatment? In your opinion, what is the best time to initiate treatment in crowded patients? Roberto Lima

Early treatment can offer many benefits to young patients when indicated. In my opinion, the best time to initiate treatment in crowded patients is after the permanent central incisors and the first permanent molars have erupted. If possible, the permanent lateral incisors should also present radiographically at least some of their roots developed. Rapid maxillary expanders and mandibular Schwarz appliances are routinely used in our practice to improve crowding during early treatment. Special attention is paid to the attached gingiva, especially on the facial of the mandibular incisors. In certain cases where crowding is severe, serial extractions may be considered, or if orthopaedic expansion is carried out, patients and parents are made aware that bicuspid extractions may still be necessary during the second phase of treatment, even in the absence of crowding, to achieve an ideal functional occlusion (usually in high angle cases).

Orthopaedic expansion during early treatment has always been considered an important tool in attaining aesthetically pleasing smiles in our practice. It has become even more important in our early treatment planning with the current increase in awareness of problems associated with sleep apnea.^13^

## What is your protocol for finishing your cases? Do you use positioners? Roberto Lima

A Centric Relation Tooth Positioner is another important tool that is used in our practice during the retention phase to produce results that are consistently optimal and excellent. The CR mounted models gathered for the fabrication of the tooth positioner also aid us in making a final evaluation of the positions of each tooth and the degree of any remaining CO-CR discrepancy. It is important to clarify that tooth positioners should not be used to correct grotesque imperfections or obvious occlusal or skeletal discrepancies that may be due to inadequate diagnosis or ill treatment mechanics. They are used in our practice during the "settling" stage to improve minor discrepancies associated with rotations, open contacts, minor marginal ridge discrepancies, and occlusal interferences that may not be readily noticed during a clinical evaluation.

When it is decided that the patient is ready to be debonded, a thorough explanation of each retainer, including the tooth positioner, is given to the patient and the accompanying parent. The reason and importance of each retainer is explained in detail using samples of each appliance and it is confirmed that the patient will be committed to wear all retainers as indicated. Over the past 18 years of using CR tooth positioners, I have found very few patients who refuse to wear one. I strongly believe that excellent communication can yield patient's trust and desirable cooperation leading to ideal treatment results.

An obvious difference in the quality of the finished results can be observed between the time of debond and after 8 weeks of wearing a CR tooth positioner.

## What is your protocol for retention? What type of retainer do you use in the lower arch? Do you believe in long-term retention? Roberto Lima

As mentioned before, our retention protocol after comprehensive treatment includes a CR tooth positioner delivered at the debond appointment (worn full-time for 4 days, and 4 hours plus nights for 7 weeks), followed by a maxillary clear retainer and a mandibular spring aligner. The upper and lower removable retainers are worn every night as soon as they are delivered. If TMD and/or parafunctional habits were present at the start of treatment, a CR splint becomes the maxillary removable retainer of choice.

After approximately 24 months of supervised retention, patients are "graduated" from our practice, but we let them and the referring dentists know that they can contact us if problems would arise in the future. We used to tell patients at this point of their retention phase that they could start wearing the removable retainers 1-2 nights per week, but we found out that some would stop wearing them all together. So instead, we now recommend wearing the retainers every night for an indefinite period of time.

If implants are necessary to replace missing teeth in a growing patient, or additional skeletal growth is being monitored, such as in Class III cases, we will continue to periodically evaluate these patients until growth has ceased (18-20 years old in females and 21-22 years old in males).

## Do you employ the indirect bonding technique in all your patients? What are the advantages of this technique? Roberto Lima

I have been using the Indirect Bonding Technique for over 24 years. It is utilized on almost every single patient in our practice. Few exceptions include some very limited early treatment cases. This technique offers a method that can be used to place brackets with repeated accuracy, minimizing the amount of bracket repositioning necessary to finish treatment in a minimal amount of time, while meeting all the goals of a functional occlusion.

No amount of detailing of an archwire can compensate for a series of poorly placed brackets. Unlike an intra-oral approach, patient's models can be viewed in all three dimensions to facilitate accurate bracket placement. Bracket placement should be pre-planned just like other aspects of the treatment plan. Indirect bonding also allows the doctor's time to be used efficiently and effectively in the clinic.

While many orthodontists may consider the Indirect Bonding Technique to be too time consuming, involving extra steps gathering impressions of the teeth and creating transfer trays in the laboratory, I believe that, at the end, this extra time spent is justified by the time saved when finishing treatments on time or ahead of time due to better bracket placement right from the start. Having done thousands of cases indirectly has allowed me the ability to recognize ill bracket positions more easily and to improve these early in treatment and therefore, avoid round tripping of the teeth.

Another area that may keep orthodontists away from using the Indirect Bonding Technique as well as mounting their cases, involves organizational skills in the clinic and throughout their practice. I believe in systems to keep any type of business flowing smoothly and to consistently obtain ideal results. A good source that can be used as reference in creating systems in any small business is the popular book by Michael Gerber, "E-Myth". In this book, he explains how one of the keys to a successful business is creating a business development cycle "Innovation, Quantification, and Orchestration." Implementation of systems and keeping things well organized and consistent is also important in any orthodontic practice. For example, in our office, we strive to keep every unit and drawer stocked the exact same way to increase efficiency, and we try to schedule most indirect bondings (start and debond appointments) with one or two technicians that are highly specialized in performing these procedures, even though the rest of the staff is also trained to perform any of them whenever necessary. This scheduling system plan allows us the ability to streamline and solve inaccuracies or failures more easily.

I would like to thank you for the invitation to be interviewed. Brazil is very dear to me, and I have become very fond of this country and its people over the years, especially after having had the opportunity to lecture in several of its beautiful cities in 2002.
